# Supplementary material for: Methods for evaluating unsupervised vector representations of genomic regions
Source: NAR Genom Bioinform. 2024 Aug 10;6(3):lqae086. doi: 10.1093/nargab/lqae086 (PMC11316252; doi:10.1093/nargab/lqae086)
Supplement: lqae086_Supplemental_File [file lqae086_supplemental_file.pdf]

Supplementary information

Table S1. Configurations of the 15 types of embeddings.

Embedding method	Name	Dimensions (D)	Window size (W)	Learning rate (r)
Binary	Binary	690	N/A	N/A
PCA	PCA-10D	10	N/A	N/A
	PCA-100D	100	N/A	N/A
Region2Vec	5W10D-0.0250r	10	5	0.025
	5W10D-0.1000r	10	5	0.1
	5W10D-0.5000r	10	5	0.5
	5W100D-0.025r	100	5	0.025
	5W100D-0.1000r	100	5	0.1
	5W100D-0.5000r	100	5	0.5
	50W10D-0.0250r	10	50	0.025
	50W10D-0.1000r	10	50	0.1
	50W10D-0.5000r	10	50	0.5
	50W100D-0.0250r	100	50	0.025
	50W100D-0.1000r	100	50	0.1
	50W100D-0.5000r	100	50	0.5

Table S2. Universes used in the experiments.

Universe	Number of regions	F_{10}	Description
Merge (100)	287,225	0.6281	Merge regions when distance $<$ 100 bps
Merge (1k)	232,626	0.5296	Merge regions when distance $<$ 1k bps
Merge (10k)	40,498	0.1990	Merge regions when distance $<$ 10k bps
Tiling (1k)	426,964	0.4552	Each region has 1k bps
Tiling (5k)	251,084	0.2659	Each region has 5k bps
Tiling (25k)	100,053	0.1672	Each region has 25k bps
DNAse Hypersensitive Sites	561,580	0.4513	External universe

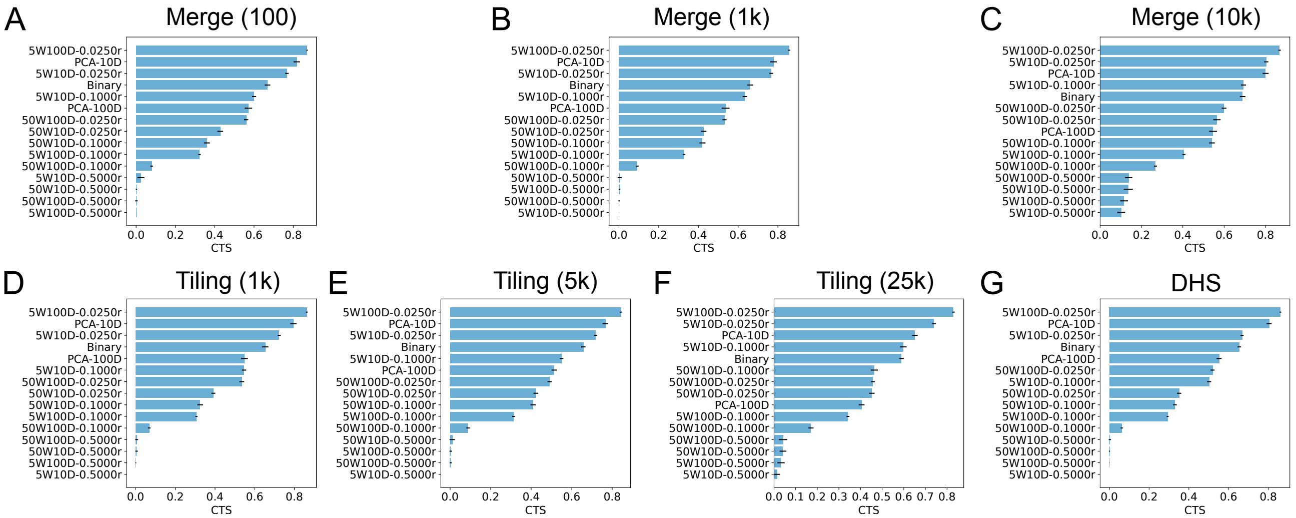

Figure S1. CTS scores of different sets of embeddings for regions in the seven universes. *Each blue horizontal bar and the associated black bar indicate the average and standard deviation of the CTSs from 20 runs of the CTS calculation for each set of region embeddings. W: context window size, D: embedding dimension, r: initial learning rate.*

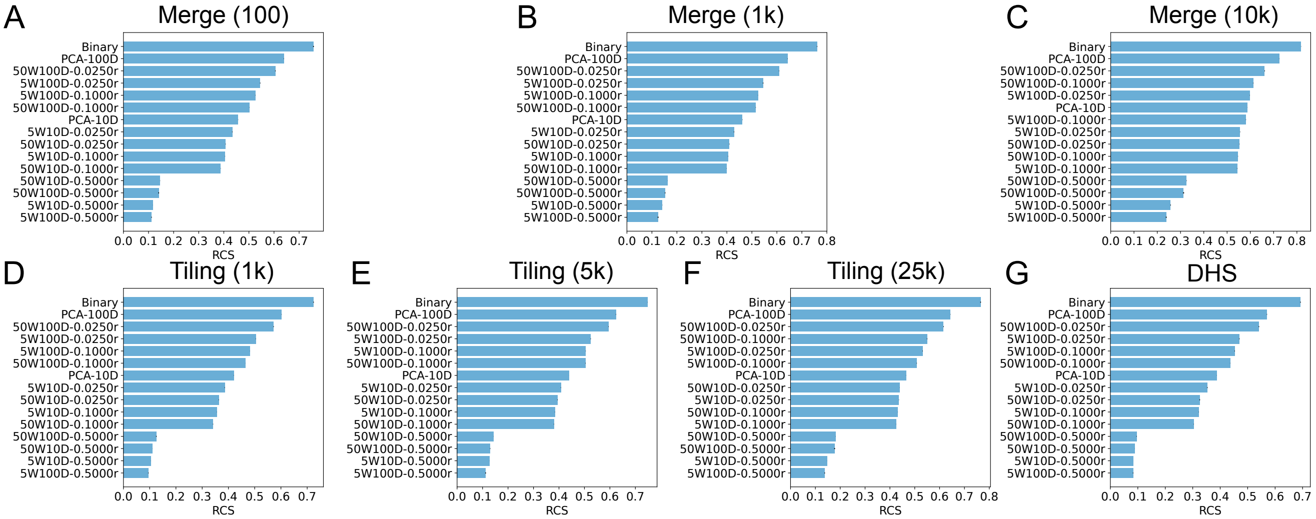

Figure** S2. **RCSs of different sets of embeddings for regions in the seven universes. *Each blue horizontal bar and the associated black bar indicate the average and standard deviation of the RCSs calculated with 5 different random seeds for each set of region embeddings. W: context window size, D: embedding dimension, r: initial learning rate.*

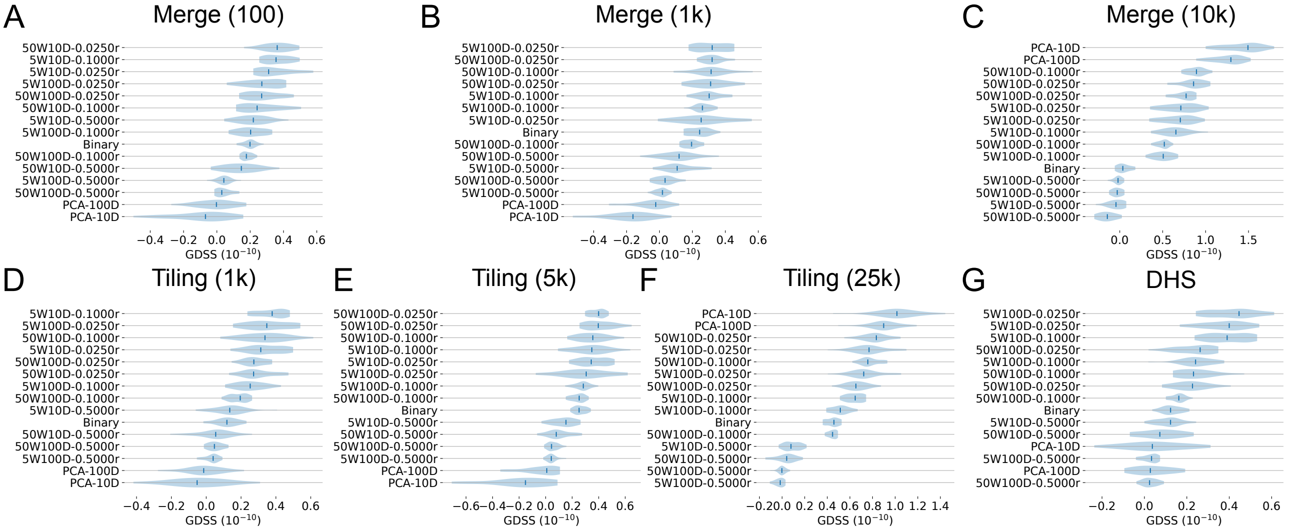

Figure** S3. **GDSSs of different sets of embeddings for regions in the seven universes. *Each blue horizontal bar and the associated black bar indicate the average and standard deviation of the GDSSs calculated with 20 different random seeds for each set of region embeddings. W: context window size, D: embedding dimension, r: initial learning rate.*

***Table** S3. Numbers of the training and test BED files for the antibody type classification task.*

Split	CTCF	Pol2	c-Myc	EZH2_(39875)	NRSF	Pol2-4H8	Rad21
Train	53	31	12	8	7	7	7
Test	36	21	9	6	5	5	5

***Table** S4. Numbers of the training and test BED files for the cell type classification task.*

Split	K562	GM12878	HepG2	HeLa-S3	H1-hESC	A549	MCF-7	HUVEC	MCF10A-Er-Src
Train	90	54	46	38	34	21	10	8	8
Test	60	36	31	26	24	14	8	6	6

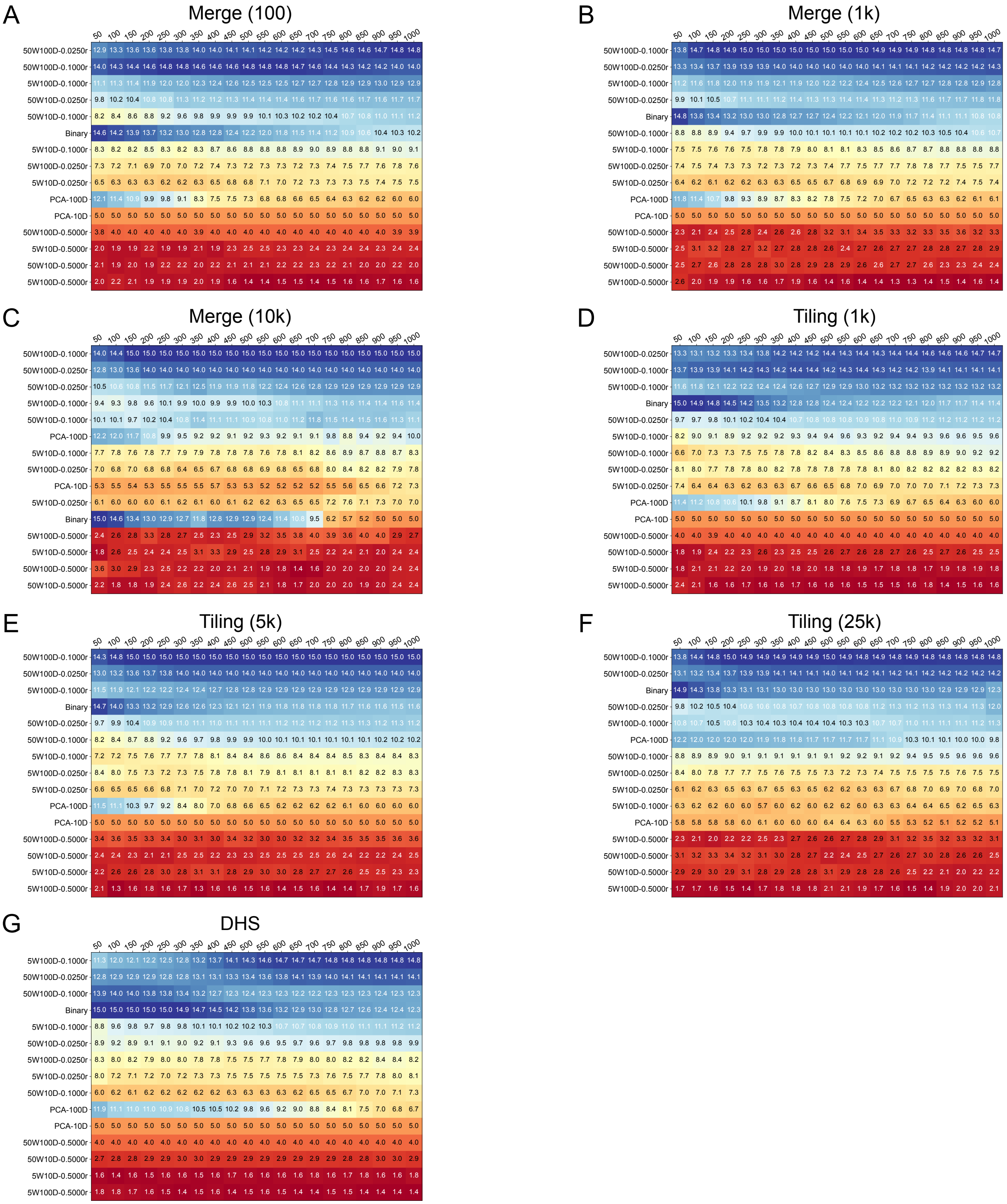

Figure S4. Average ranks of each set of region embeddings under different Us over 20 runs of the calculation of NPS. *A large rank indicates a high NPS. W: context window size, D: embedding dimension, r: initial learning rate.*

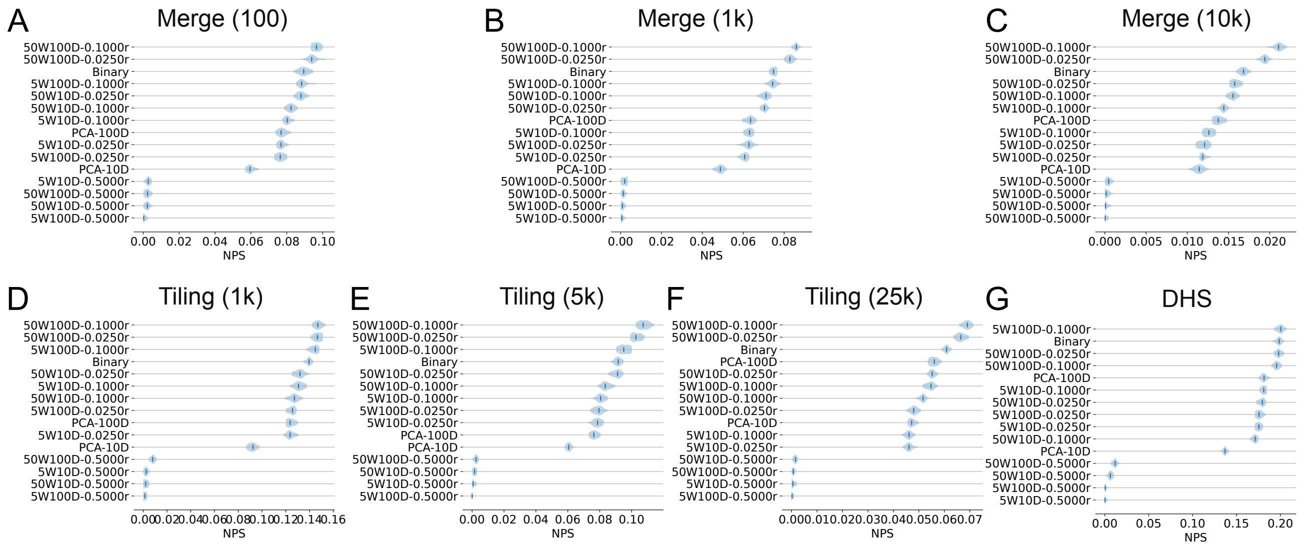

__Figure S5. NPSs of different sets of embeddings for regions in the seven universes.__ Each blue horizontal bar and the associated black bar indicate the average and standard deviation of the NPSs calculated with 20 different random seeds for each set of region embeddings. W: context window size, D: embedding dimension, r: initial learning rate.

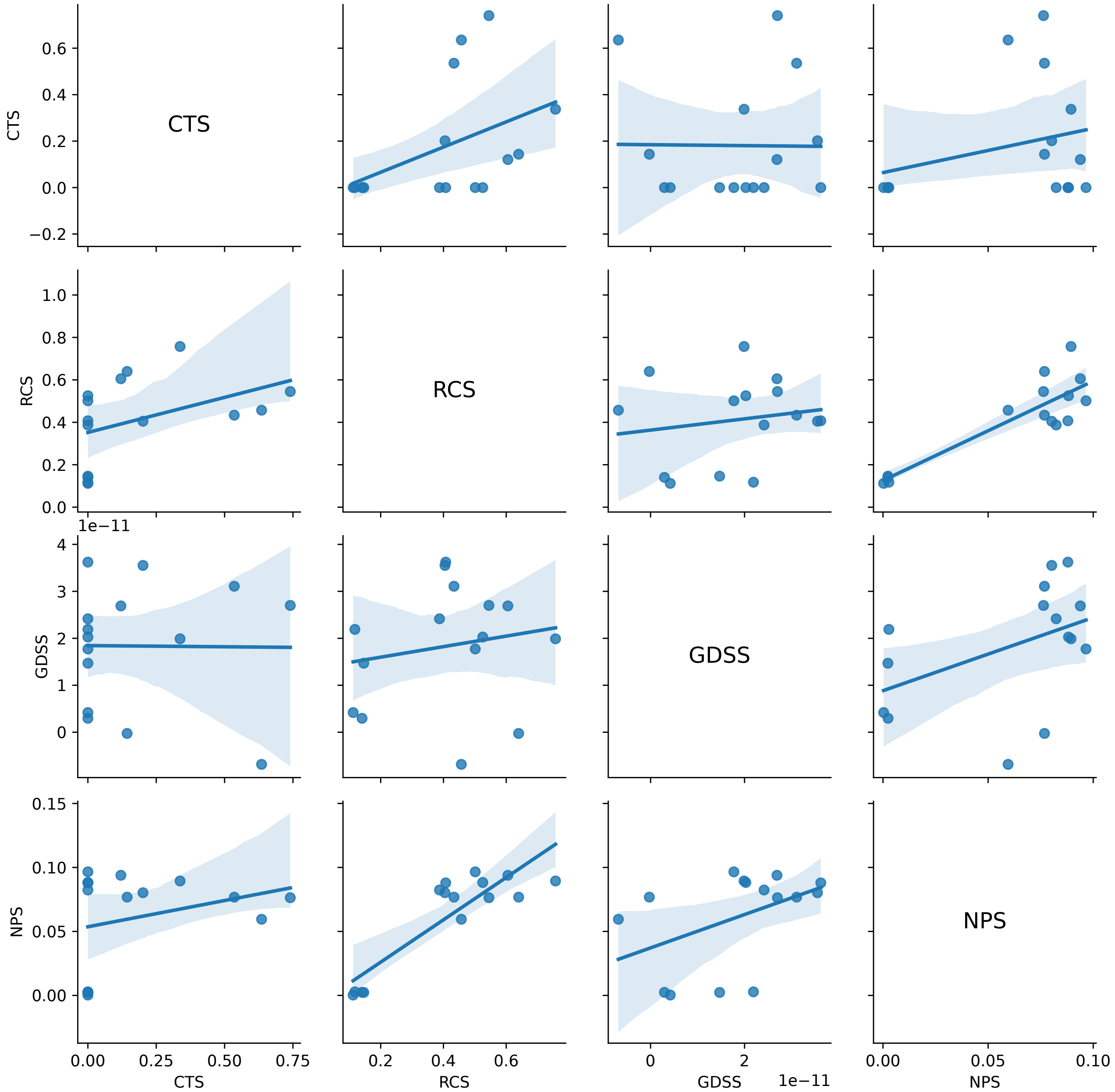

Figure S6. Pairwise correlations between the CTS, RCS, GDSS, and NPS for the Merge (100) universe.
